# Non-destructive evaluation of UV pulse laser-induced damage performance of fused silica optics

**DOI:** 10.1038/s41598-017-16467-2

**Published:** 2017-11-24

**Authors:** Jin Huang, Fengrui Wang, Hongjie Liu, Feng Geng, Xiaodong Jiang, Laixi Sun, Xin Ye, Qingzhi Li, Weidong Wu, Wanguo Zheng, Dunlu Sun

**Affiliations:** 1 Hefei Institutes of Physical Science, Chinese Academy of Sciences, Hefei, 230031 China; 2 University of Science and Technology of China, Hefei, 230026 China; 3 Research Center of Laser Fusion, China Academy of Engineering Physics, Mianyang, 621900 China

## Abstract

The surface laser damage performance of fused silica optics is related to the distribution of surface defects. In this study, we used chemical etching assisted by ultrasound and magnetorheological finishing to modify defect distribution in a fused silica surface, resulting in fused silica samples with different laser damage performance. Non-destructive test methods such as UV laser-induced fluorescence imaging and photo-thermal deflection were used to characterize the surface defects that contribute to the absorption of UV laser radiation. Our results indicate that the two methods can quantitatively distinguish differences in the distribution of absorptive defects in fused silica samples subjected to different post-processing steps. The percentage of fluorescence defects and the weak absorption coefficient were strongly related to the damage threshold and damage density of fused silica optics, as confirmed by the correlation curves built from statistical analysis of experimental data. The results show that non-destructive evaluation methods such as laser-induced fluorescence and photo-thermal absorption can be effectively applied to estimate the damage performance of fused silica optics at 351 nm pulse laser radiation. This indirect evaluation method is effective for laser damage performance assessment of fused silica optics prior to utilization.

## Introduction

As the most commonly used ultraviolet (UV) optic material in large high-power laser facilities such as the National Ignition Facility in the United States, the Laser MegaJoule in France, and the ShenGuang (SG)III laser facility in China, transparent fused silica optics have many advantages, such as low absorption at UV wavelength, high chemical stability, and excellent anti-damage performance^1–4^. Low fluence (energy density of the pulse laser) damage precursors, such as surface polishing-induced defects, can limit laser damage performance of fused silica optics. To improve the laser damage performance of fused silica optics, significant efforts have been made to improve polishing processes and utilize different kinds of post-treatment processes to mitigate polishing-induced defects^5–8^. The laser damage performance test is necessary to assess the manufacturing process and evaluate product quality prior to utilization. For small size optics with centimeter-sized apertures, a damage threshold test of S on 1 and R on 1 is generally used to estimate laser damage performance. Larger size optics require testing the damage density using a raster-scan method, which provides a bigger sampling area and provides results with-statistical significance^9–12^. However, these laser damage tests are destructive, and result in losing part or all of the function of tested optics, making these methods costly, particularly for big aperture optics. Therefore, it is important to find a new effective and non-destructive method to evaluate laser-induced damage of fused silica optics.

Significant efforts have been made to evaluate the influences of fused silica surface defects on laser damage performance. Two main kinds of defects are responsible for inducing laser damage of fused silica^13–15^, absorptive contaminants (e.g., Ce and La) in the Beilby layer from polishing and sub-surface damage (SSD) from grinding and/or polishing of brittle material surfaces. These precursors could decrease the laser-induced damage thresholds by directly absorbing UV laser energy, reducing the mechanical strength, or providing a highly enhanced local optical field. Further experimental studies showed that inorganic salt containing elements of K, Na, Ca, Mg and organic contaminants can limit fused silica optic damage performance in high laser fluence above 20 J/cm^2^, defined as high fluence laser damage precursors^16,17^. Together, this research suggested that laser damage performance of fused silica surface is strongly affected by defects. Therefore, laser damage performance of fused silica optics can be indirectly evaluated by defect characterization, which can partly replace the traditional destructive laser damage performance test. However, because of improved defect mitigation technology, such as AMP (advanced mitigation process)^18^, defect types directly related to damage performance have changed. For example, low fluence laser damage precursors such as Ce impurity have been controlled remarkably. With increased damage performance of fused silica, high fluence laser damage precursors are now more important factors that restrict fused silica damage performance. Although the crucial defect types related to damage performance have changed with improved defect mitigation technology, the initiation of laser damage still originates from the absorptive properties of defects, which actively respond to laser radiation^19,20^. Therefore, by characterizing the absorptive defects of fused silica surface and clarifying the influence of defect parameters on the damage performance of fused silica, we can realize non-destructive evaluation of laser-induced damage performance for fused silica optics.

In this work, by using ultrasound-assisted chemical etching and magnetorheological finishing, fused silica samples with different laser damage performances were obtained. To determine the relationship between absorptive defects and damage performance, laser-induced fluorescence imaging (LIFM) and photo-thermal deflection (PTD) were used to characterize the percentage of fluorescent defects and determine the weak absorption coefficient. The damage thresholds and damage density were respectively tested with a UV laser damage test system. We then analyzed the quantitative relationship of absorptive defect parameters and laser damage performance, and demonstrated the process of realizing non-destructive evaluation of laser induced damage performance by characterization of absorptive defects.

## Results

### Damage performance test results of all the samples

**Figure 1:**
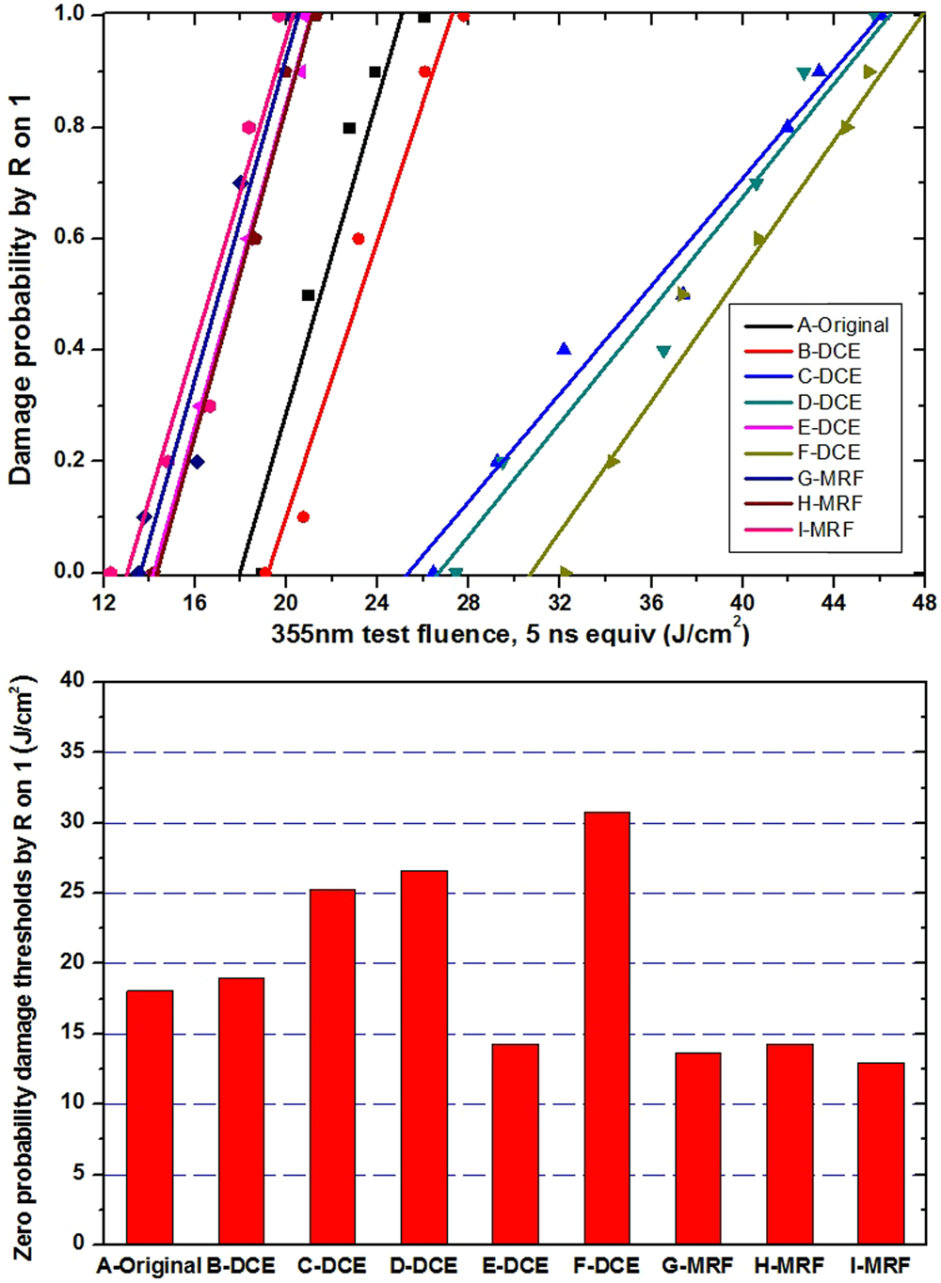
Damage probability (up) and corresponding zero probability damage thresholds (down) of different post-process samples.

Dynamic chemical etching (DCE) techniques, such as AMP, are effective methods to decrease sub-surface defects and improve laser damage performance^21–23^. Oxidizing acid and hydrofluoric (HF) acid can restrain surface contaminants, subsurface impurity elements, and passivating sub-surface damage (SSD). A compound frequency ultrasound with 40 KHz~270 Khz was used to improve etching efficiency. Magneto-rheological finishing (MRF), which is based on flexible machining, can decrease the SSD induced by mechanical polishing and is an attractive post-process method^24–26^. In the experiment, we used DCE and MRF to change the defect distribution of a fused silica sub-surface and prepared nine samples with different post-process parameters. The zero probability damage thresholds of all the samples at 355 nm pulse laser radiation were tested and are shown in Fig. 1.

Compared to the original sample (A-original), the samples after dynamic chemical etching exhibit higher damage thresholds. The degree of this increase is related to the etching time, and samples with longer etching time had higher damage thresholds. For example, the damage threshold of sample F-DCE was the highest, and the damage threshold of sample E-DCE was lowest, likely because the environment clean class of dynamic chemical etching was only level 10000. The damage thresholds of MRF samples were not improved obviously, likely related to the secondary pollution of MRF fluid residue on surfaces. Zero probability damage thresholds of all the samples shown in Fig. 1 contain a wide region of 13.2~30.8 J/cm^2^, indicating significant differences for different post-treatments.

**Figure 2:**
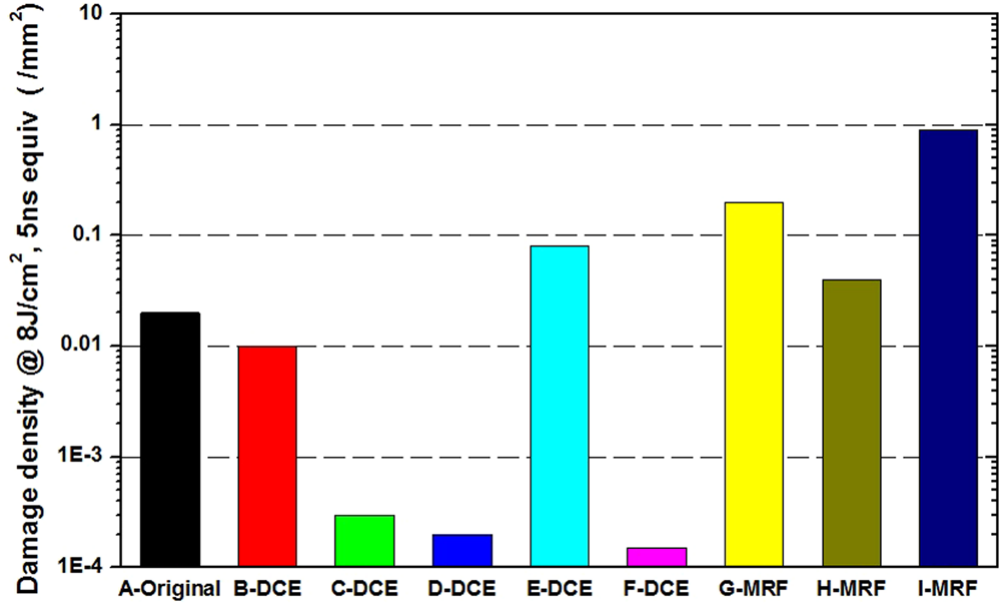
Damage density contrast of different samples at 8 J/cm^2^ fluence.

Next, we used Raster-scan to test the damage densities of all the samples. In contrast with damage thresholds, a damage density test with bigger sampling area can identify more defects present with a low probability distribution, so the test results better reflect the influence of defect distribution on damage performance. Figure 2 shows the damage density test results of all the samples. The result shows that the damage density of HF acid etching samples is two orders lower than that of the non-etched samples. Samples treated with a longer etching time exhibited lower damage density. In contrast with the pristine sample (A-Original), although MRF can decrease SSD, the damage performance of the MRF samples was worse, which indicates that the process can lead to more serious secondary pollution on sample surfaces.

The damage test results indicate that in the post-process of DCE and MRF, the damage performance of samples is related to process parameters and secondary pollution control, which change the defect status of the fused silica surface layer. These post-processes provided us appropriate samples to study the influence of absorptive defects on damage performance.

### Absorptive defect distribution in the sample surface

In order to determine the relationship between absorptive defects and damage performance, laser-induced fluorescence imaging (LIFI) and photo-thermal deflection (PTD) were used to characterize all surface absorptive defects.

**Figure 3:**
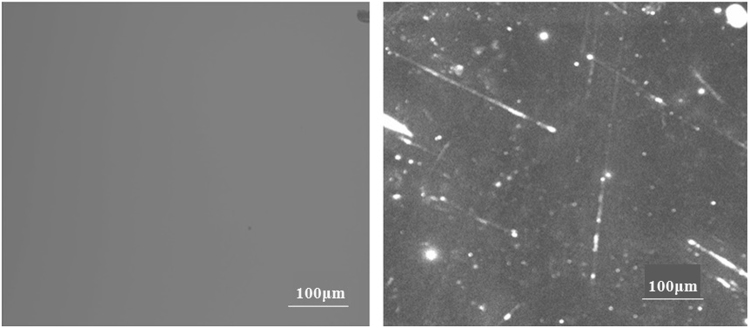
Comparison of the same position of a mechanical polishing fused silica sample surface under bright field micro imaging(left) and UV laser induced fluorescence imaging (right).

**Figure 4:**
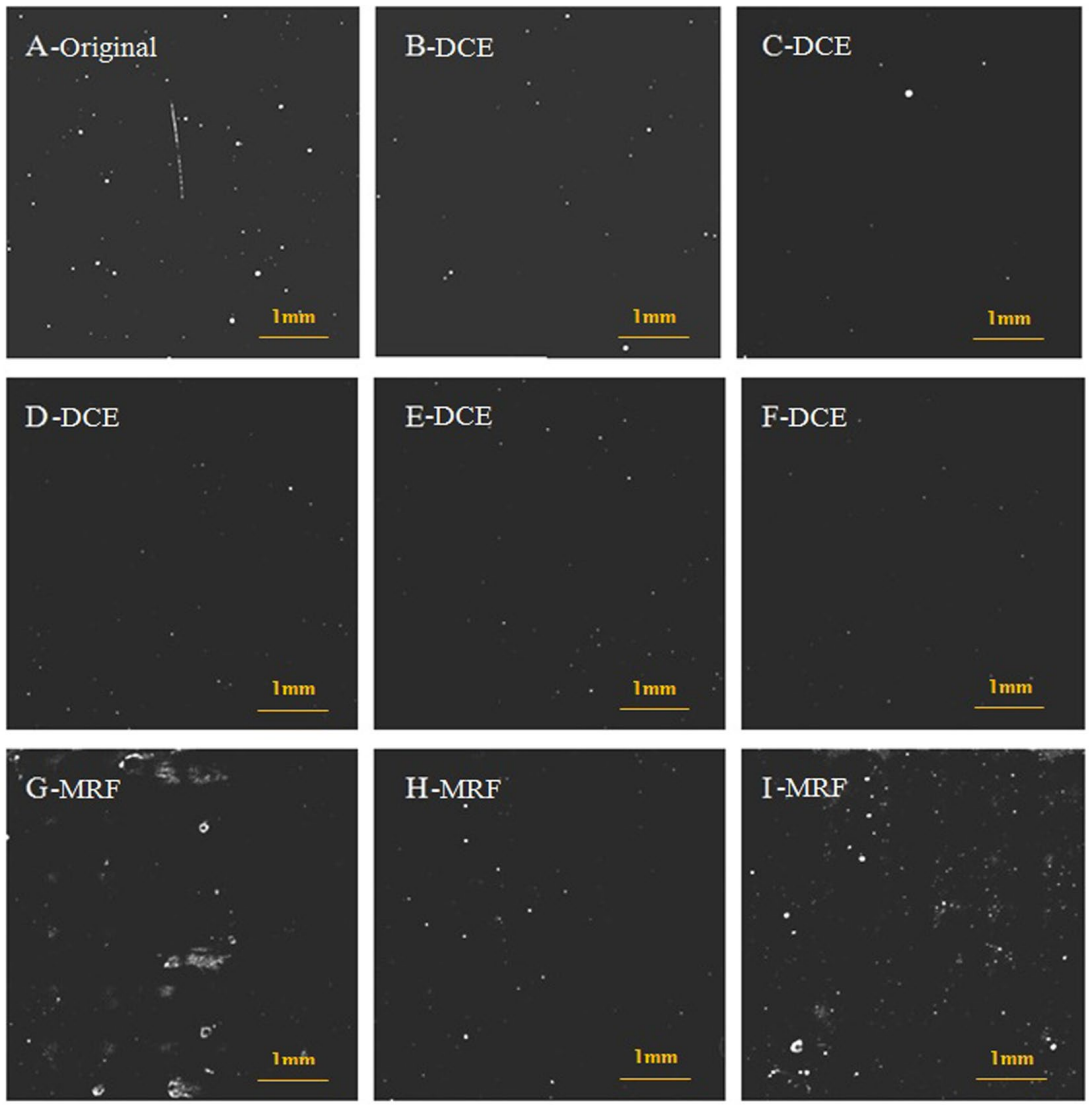
Comparison of typical fluorescence defect distribution at the sample surface treatment with DCE and MRF.

**Figure 5:**
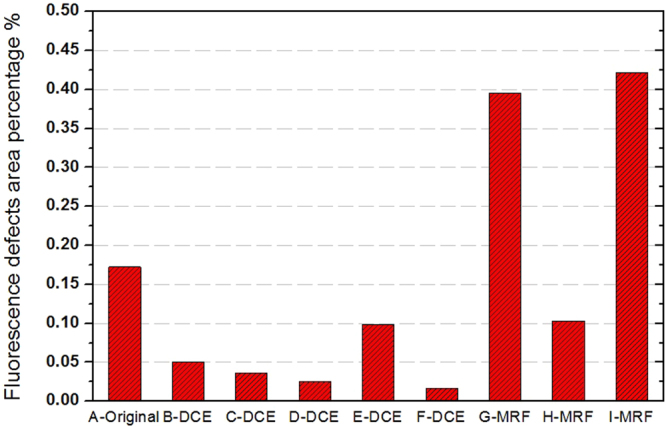
Results of fluorescence defects area percentage for the nine samples.

As a sort of non- destructive characterization method, light-induced fluorescence is an effective tool to study defects of optical material^27–31^.Although fluorescence objects will not produce an obvious heating effect after absorbing laser energy, based on the fluorescence mechanism of absorption-transition-emission, these fluorescent defects still can partly reflect the absorption characteristic of surface defects. In particular, LIFI is used to effectively characterize SSD and polishing-induced point defects of fused silica optics surface^14,15,17^ and identify some laser-induced damage precursors. Figure 3 shows the same position of a mechanical polished fused silica sample surface under bright field micro imaging and fluorescence imaging excited by a 375 nm laser. We can see that the fluorescence imaging reveals numerous defects that are invisible in bright field micro imaging. The line type fluorescent objects indicate polishing-induced scratches and cracks, and the spot objects are polishing-induced point defects, such as pit holes and polishing residual gathering. Figure 4 presents the typical results of a UV laser induced fluorescence image of this experiment samples. The results indicate that LIFI can reflect the influences of dynamic chemical etching and MRF on the sub-surface defects of fused silica optics. For the chemical etching samples, the original sample surface showed numerous scratches and point defects. After oxidization cleaning and HF acid etching, the quantity of fluorescence defects was decreased obviously. The sample treated with 100 min etching time (F-DCE) had the least fluorescence defects. The MRF samples have many more fluorescent defects. Averages of fluorescence defects area percentage of all the samples are shown in Fig. 5.

**Figure 6:**
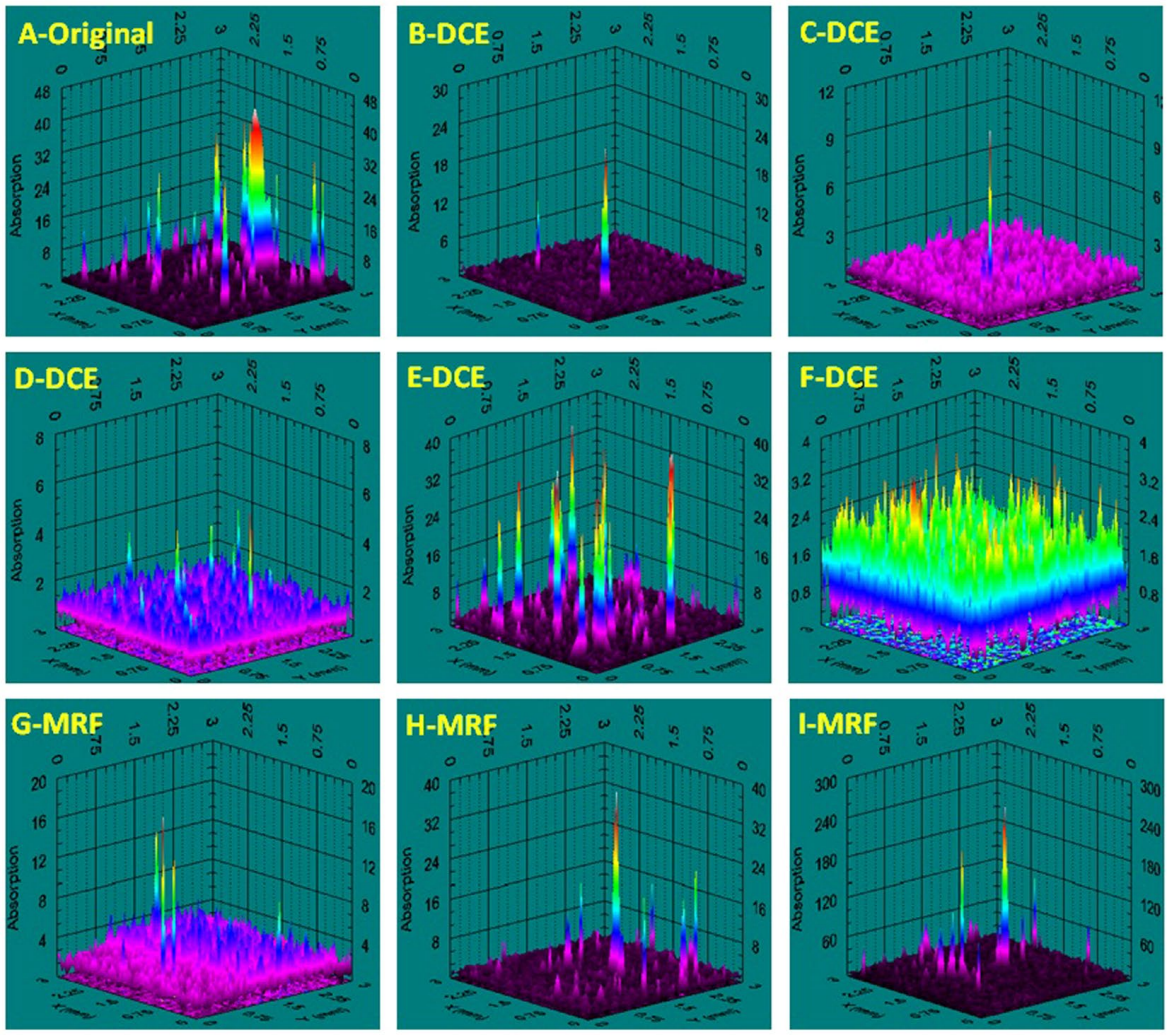
Comparison of typical 355 nm laser absorption distribution on sample surface after dynamic chemical etching and MRF.

**Figure 7:**
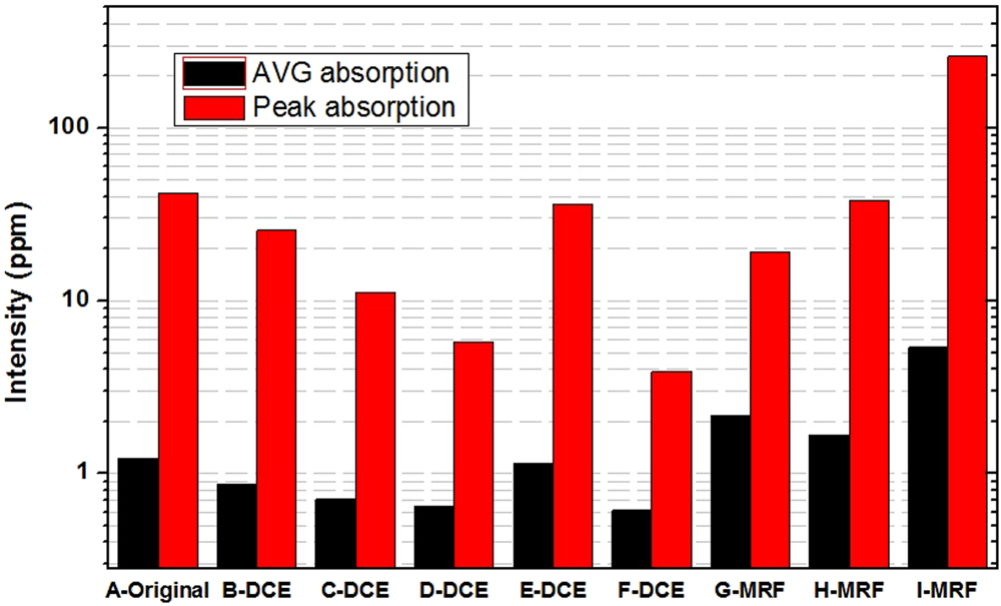
Quantitative 355 nm absorption results for all samples.

Photo-thermal deflection technique is a relative measurement of laser weak absorption for transparent optical material^32–35^, the most sensitive optical material absorption test. Figure 6 shows the 355 nm laser absorption distribution for all nine samples surfaces of this experiment. The result indicates that weak absorption test data reveals the absorptive defect distribution character of the tested region, including average absorption and peak (maximum) absorption. Average absorption is shown as the flat bottom in three-dimensional absorption distribution figures and includes most data points. The average absorption values reflect the high density intrinsic absorption of surface defects, such as surface metal impurities with uniform distribution. Peak absorption is shown as individual highest peak in three-dimensional absorption distribution figures. These peaks reflect low density strong absorption defects in sample surfaces, such as SSD and point defects. Figure 7 shows the absorption statistic results for all samples. The results indicate that the surface post-process can effectively change the 355 nm laser absorption coefficient of the fused silica surface. For the samples treated by oxidization cleaning and longer HF acid etching time, the peak absorption decreased from 40 ppm to several ppm, and strong absorption defects were eliminated. The average absorption value of the original sample (A-original) was about 1.2 ppm, was reduced to 0.9 ppm after oxidization cleaning (B-DCE), and was further reduced to 0.6 ppm with increased HF etching time. The absorption of sample E-DCE is at the level of original sample, which might be related to a lower environment clean class in dynamic chemical etching. The surface absorption of all MRF samples was increased obviously. Overall, the UV-absorptive levels of the nine samples were different obviously, indicating that DCE and MRF changed the distribution of absorptive defects in fused silica surface.

## Discussion

To achieve non-destructive evaluation of laser-induced damage performance of fused silica, the relationship between surface defect distribution and laser damage performance must be determined. MRF can remove SSD by flexible polishing, but residual magneto-rheological fluid makes it easy to introduce new secondary pollution and increase impurity elements on the surface, such as Fe. Dynamic chemical etching can obviously decrease the impurities and SSD on optics surface by peeling off the polishing layer and ultra clean rinsing. The experimental results indicate that MRF and dynamic etching can effectively change the distribution of fluorescent defects and absorptive defects, resulting in obvious changes in the laser damage thresholds and damage density at 355 nm pulse laser. Overall, fluorescent defects and absorptive defects have strong influences on laser damage performance of the fused silica surface, allowing non-destructive evaluation of damage performance by defect characterization. It should be noted that there is no specific numerical distribution characteristic for defect parameter (*x*_*i*_) and damage performance (*y*_*i*_), but a rank correlation exists between such two variables. Thus, the Spearman rank correlation function can be used to estimate the correlation coefficient between defect distribution and damage performance of fused silica optics. The Spearman rank correlation coefficient is calculated according to the following equation^36^:

1 $$r(s)=\frac{1-6\sum _{i=1}^{n}{{d}_{i}}^{2}}{{n}^{3}-n}$$

where *d*_*i*_ is the difference between ranks for each *x*_*i*_, *y*_*i*_ data pair and *n* is the number of data pairs. The calculated results are shown in Table 1.

**Table 1:** Spearman rank correlation coefficient between defect distribution parameters and damage performance of fused silica optics.

Defect distribution parameters	Damage thresholds (0%)		Damage density at 8 J/cm^2^	
	Correlative coefficient	Sig	Correlative coefficient	Sig
Fluorescence defects area percentage	−0.946	1.170E-4	0.933	2.359E-4
Average absorption	−0.971	1.374E-5	0.951	8.762E-5
Peak absorption	−0.778	0.014	0.767	0.016

The Spearman correlation coefficient is used to estimate the monotonic increasing or decreasing relationship between two variables. When the value is 1, the relation of two variables is monotonic increasing; when the value is −1, the relation of two variables is monotonic decreasing; and when the value is 0, the two variables are completely uncorrelated. The value of Sig indicates the reliability of the correlation analysis results. A smaller value of Sig means higher reliability, which should be less than 0.01 in the analysis. The results shown in Table 1 show that the fluorescence defect percentage and average absorption are negatively correlated with zero probability damage thresholds, and have a positive correlation with damage density. The absolute values of correlation coefficient are greater than 0.9. Compared to the fluorescent defects percentage, there is better correlation between the average absorption and damage performance. On the other hand, the correlation coefficient between peak absorption and damage performance is much lower, and the Sig value is greater than 0.01. Although the peak absorption indicates the strongest absorptive point defect that can induce laser damage in the tested region, the distribution probability of these low fluence laser damage precursors is usually very low. So, for a damage threshold test based on limited random sampling, the peak absorption defects could not be detected in a test. For the damage density test, the test results depend on the defects presenting at a high distribution probability. Because there are fewer damage points induced by peak absorption defects, there is little influence on damage density. In contrast, the fluorescent defect percentage and average absorption may statistically reflect the total level of surface defects present at higher distribution probability. Thus, these defects mostly determine damage density and damage thresholds, so the correlation coefficients with damage performance are much better. Overall, the use of fluorescent defect percentage and average absorption with statistical significance to evaluate damage performance of fused silica is a more suitable effective approach.

### Non-destructive evaluation of zero probability damage threshold of fused silica

**Figure 8:**
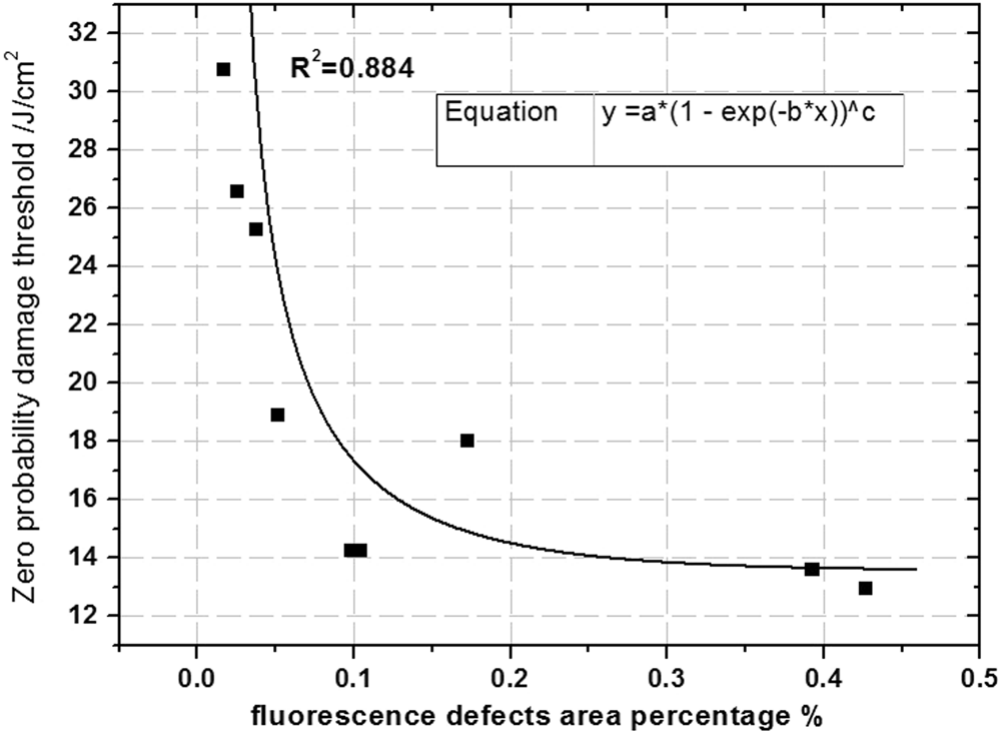
Fitting relationship between the fluorescence defect percentage and zero probability damage thresholds.

Correlation analysis results of Table 1 indicate that the fluorescent defect percentage and the average absorption have a high correlation with zero probability damage thresholds. Therefore, we can use a fitted curve to determine the quantitative relationship between these two variables, as shown in Fig. 8. The X axis is the fluorescence defects percentage of the nine samples, and the Y axis is the zero probability damage threshold of these samples. The Exponential-Chapman function was used for the fitting and the R^2^ is 0.884.

The fitting result shows that there is an exponential attenuation relationship between the fluorescent defect percentage and the zero probability damage threshold. The degree of fitting in high damage thresholds (greater than 19 J/cm^2^) is better than that of low damage thresholds, which implies that surface defect types are more varied for low damage threshold fused silica, and fluorescent defects are not the only factor that determines zero probability damage thresholds. Considering the fitting degree, a fluorescent defect percentage is appropriate to evaluate the zero probability damage threshold of fused silica.

**Figure 9:**
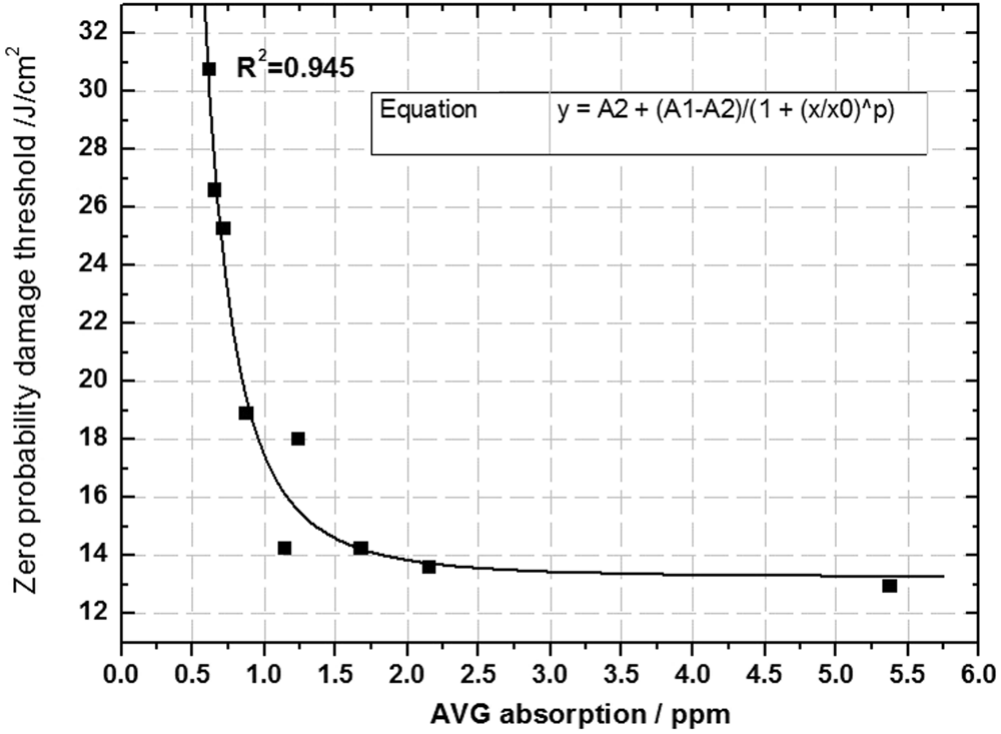
Fitting relationship between average absorption and zero probability damage thresholds.

We plotted the surface average absorption of the nine samples as the X-axis and zero probability damage thresholds as the Y-axis. Quantitative fitting curve between the surface average absorption and zero probability damage thresholds is shown in Fig. 9.

The fitted curve shows that there is still an exponential attenuation relationship between the surface average absorption and the zero probability damage threshold. When the average absorption is below 1 ppm, the damage threshold decreases quickly with the increase of the average absorption. This relationship weakens for average absorption over 1ppm, which indicates that fused silica optics with high damage thresholds are more sensitive to extrinsic absorption defects. The fitted curve was obtained using Exponential-Logistic function and degree of fitting coefficient (R^2^) is 0.945, so we can use the surface average absorption to indirectly evaluate the damage threshold with high reliability.

### Non-destructive evaluation of damage density of fused silica

**Figure 10:**
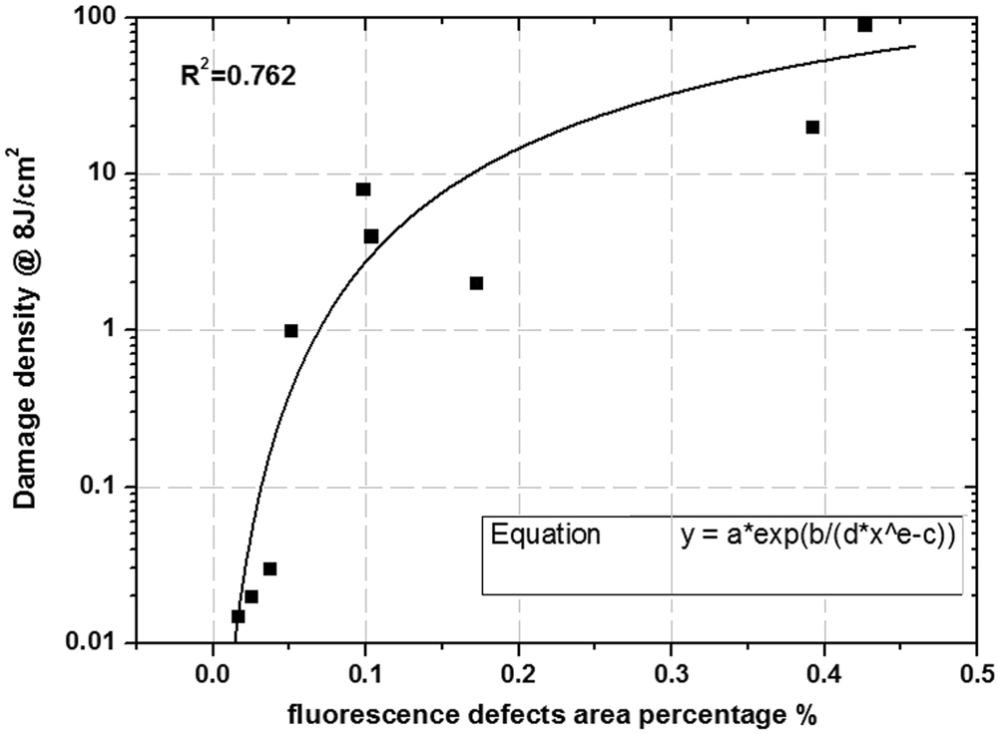
Fitting relationship between fluorescence defect percentage and damage density at 8 J/cm^2^ test fluence.

Based on the correlation analysis result of Table 1, the fluorescent defect percentage and average absorption still showed a high correlation with damage density, with Spearman coefficients of 0.933 and 0.951, respectively. This strong correlation behavior indicates that the same method can be used to indirectly evaluate damage density. Figure 10 shows the quantitative fitting curve between fluorescence defect percentage and damage density at 8 J/cm^2^ test fluence.

The fitted result shows that there is an exponential growth relationship between the two variables, but the fitting coefficient is just 0.762, due to some distorted data points in the high damage density range (greater than 1/cm^2^). These distorted points demonstrate that fluorescent defects do not completely reflect the damage density of fused silica optics with low damage performance. Clearly, there are other types of defects affecting the damage density of fused silica, resulting in a weak correlation of fluorescent defects on damage density. This is similar to the influence of fluorescent defects on zero probability damage threshold in low threshold fused silica optics.

**Figure 11:**
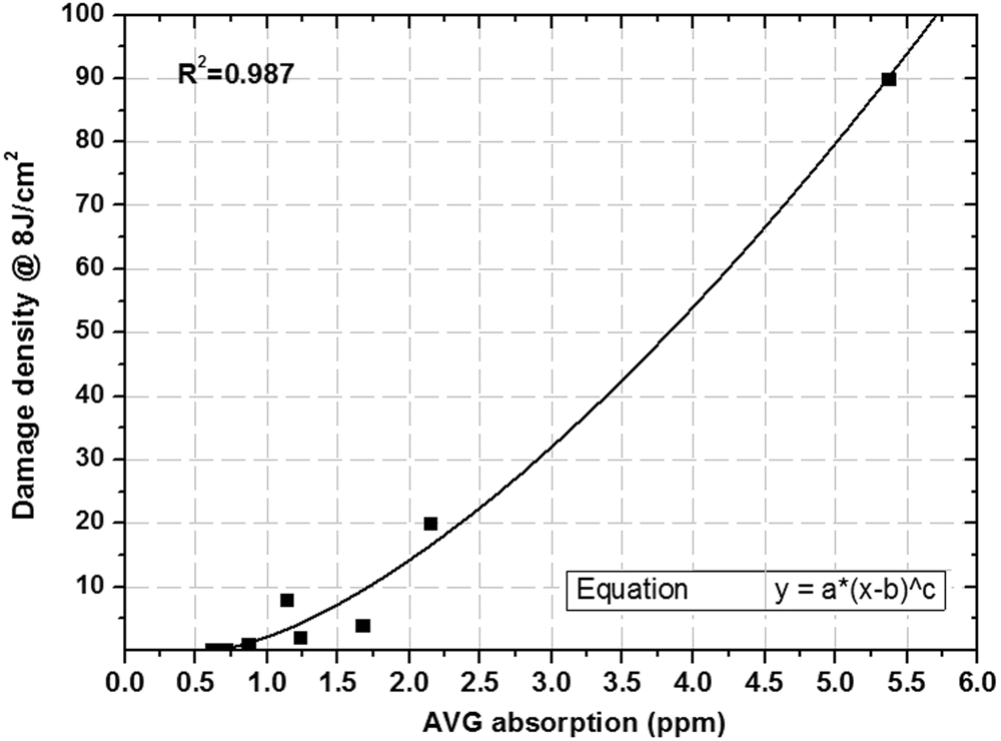
Fitting relationship between 355 nm average absorption and damage density at 355 nm 8 J/cm^2^ test fluence.

Surface average absorption is the other defect characterization parameter used to evaluate the damage density of fused silica. Figure 11 shows the quantitative fitting curve between the surface average absorption and damage density at 8 J/cm^2^ test fluence.

**Figure 12:**
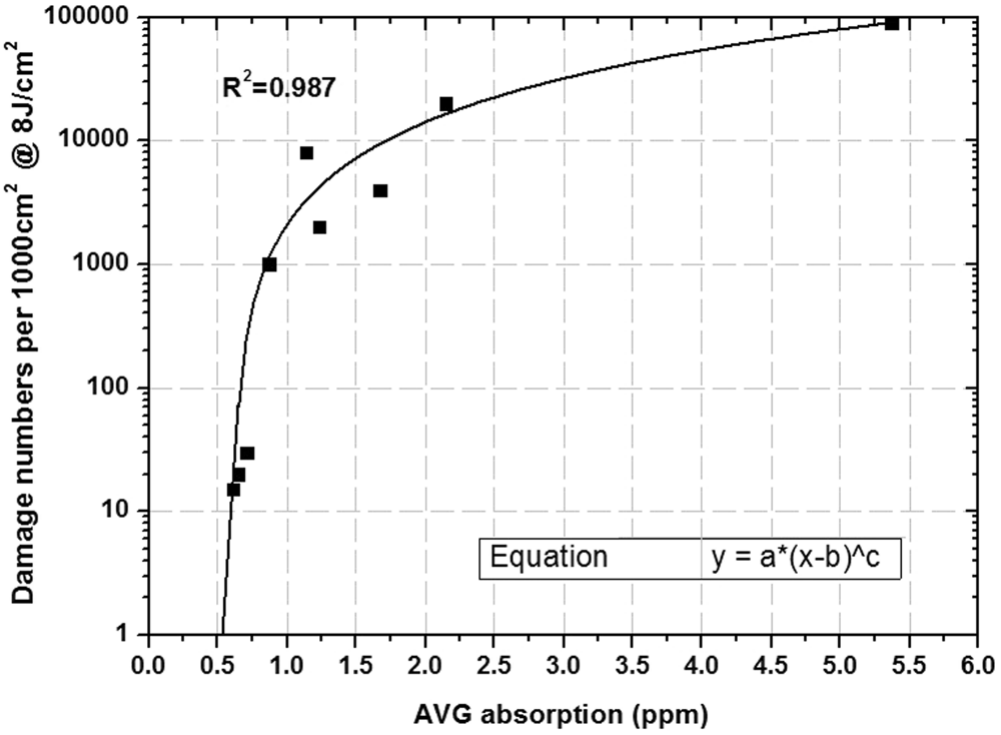
Fitting relationship between 355 nm average absorption and damage point numbers of 1000 cm^2^ fused silica optics at 8 J/cm^2^ test fluence.

In Fig. 11, the relationship of average absorption and damage density is fitted well by a power function. The fitted coefficient is 0.987, which indicates that the fitted curve can accurately reflect the relationship between average absorption and damage density. The fitted result shows that the damage density of fused silica is affected by the surface average absorption, which directly contributes to laser damage. For large aperture fused silica optics in large high-power laser facilities, we need to forecast damage point numbers of whole optics at the target operation fluence. For example, for large size fused silica optics with 1000 cm^2^ and 8 J/cm^2^ operation fluence at 351 nm wavelength, the relation curve of damage point numbers of whole optics and surface average absorption is shown in Fig. 12.

The results show that when the average absorption is below 1 ppm, tiny changes of absorption value can affect the damage density with orders of magnitude, but the influence weakens when the average absorption is over 1 ppm. When surface absorptive defects gather and induce a high average absorption level, the influence of absorption characteristics on damage density will weaken. Therefore, for fused silica surface with a higher average absorption level, it is difficult to decrease the damage density by an order of magnitude by reducing the absorption. In order to control damage point numbers in whole optics, the average absorption coefficient must be controlled at a low level (less than 1 ppm). With this condition, we can achieve damage density reduction by orders of magnitude by further decreasing absorption.

We can evaluate the 8 J/cm^2^ fluence damage density of large size fused silica optics in high-power laser facilities because there is a monotonic increasing relationship between damage density and test laser fluence. This method is also suitable for damage density evaluation at other laser fluence. In addition, according to the relationship curve, we can determine specifications for average absorption for a given damage performance level. For example, in order to insure that fused silica optics with a 1000 cm^2^ aperture does not contain any damage points at 8 J/cm^2^ operation fluence, the average absorption coefficient should be controlled under 0.5 ppm. It must be clarified that the quantitative relationship between defects and damage performance is not constant, and can be affected by damage test methods, defect test methods, and test equipment status.

## Conclusions

We present a method using absorptive defects as a non-destructive technology to evaluate laser-induced damage performance of fused silica optics at 351 nm pulse laser. The conclusions of this research are as follows:

A. Percentage of fluorescent defects has an exponential attenuation relationship with zero probability damage thresholds and an exponential growth relationship with damage density. The fitted relation is more suitable for fused silica optics with high damage performance, and the reliability is not sufficient to evaluate damage performance of fused silica with poorer surface quality.
B. There is no significant correlation between peak absorption and laser damage performance, indicating that peak absorption is not a useful evaluation parameter of fused silica damage performance.
C. The average absorption coefficient has a significant correlation with zero probability damage threshold and damage density. Quantitative fitting curves illustrate the exponential attenuation relationship and power exponent growth relationship. All the fitted coefficients are over 0.9, indicating its usefulness as an evaluation parameter of fused silica damage performance.

In summary, we can indirectly evaluate the damage threshold and damage density at 351 nm pulse laser by charactering the absorptive defects in fused silica surface. Although the accuracy of this method is not comparable with that of the traditional laser damage test, this is a non-destructive evaluation method of damage performance that can be used for UV laser damage performance pre-judgment of fused silica optics prior to utilization.

## Materials and Methods

### Sample preparation

Nine100 mm × 100 mm size, 10 mm thick Heraeus Suprasil 312 fused silica samples were incised. In the experiment, all samples were finished using CeO_2_ polishing powder of the same batch and from the same vendor, and then were cleaned by 1% concentration hydrofluoricacid. We used DCE and MRF technologies to change the defect distribution of the fused silica sub-surface. Nine samples with different post-process parameters were obtained, as listed in Table 2.

**Table 2:** Surface post-process parameters of the nine samples.

Name of sample	Pre-process	Parameters of DCE and MRF	Operating air environment
A-Original	mechanical polishing by CeO_2_, cleaned by 1% concentration HF acid.	/	/
B-DCE		oxidization clean (HNO_3_:H_2_O_2_ = 3:1).	Class100, Ultrasound
C-DCE		oxidization clean and HF(HF:NH_4_F:H_2_O = 1:4:10) etching 5 min	Class100, Ultrasound
D-DCE		oxidization clean and HF (HF:NH_4_F:H_2_O = 1:4:10)etching 50 min	Class100, Ultrasound
E-DCE		oxidization clean and HF (HF:NH_4_F:H_2_O = 1:4:10)etching 50 min	Class10000, Ultrasound
F-DCE		oxidization clean and HF (HF:NH_4_F:H_2_O = 1:4:10) etching 100 min	Class100, Ultrasound
G-MRF		MRF of 1 μm	Class100
H-MRF		MRF of 2 μm	Class100
I-MRF		MRF of 5 μm	Class100

### Characteristics

A laser scanning confocal fluorescence microscope system was used to characterize mechanical polishing defects in the surface of fused silica. This is an integrated microscope system consisting of a fluorescence microscope, laser light sources, a scan head that directs the laser on the sample and collects the emission, and a computer with software to control the scan head and display the acquisition. This system allows the simultaneous measurement of both fluorescence images and bright field images. The excited laser output power is about 50 mW with a wavelength of 375 nm. Fluorescence images were detected using a 410 nm high-pass filter with 20 X objective. We can obtain a large field image with 5 mm × 5 mm by stitching 8 × 8 small images. The number of fluorescence defects present in a region was determined using an image grayscale process. To get statistical results, five regions with 5 mm × 5 mm were selected randomly form sample surface, the defects were counted for each tested region, and the average of fluorescence defects percentage for all tested regions is as final test results.

**Figure 13:**
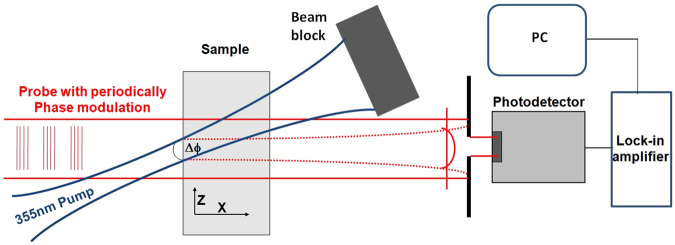
Schematic of photo-thermal deflection for weak absorption induced by laser.

A photo-thermal deflection apparatus based on a photo-thermal common-path interferometer was used to reflect the absorptive defects. The pump beam is a 355 nm quasi-continuous laser with 1 W output power and was focused on sample surface with a 60 μm focal spot (1/e^2^), and the probe beam is a modulated He-Ne laser that overlapped with the pump laser on surface of tested samples. When there are absorptive defects in the optical material surface, the pumping laser energy is partly absorbed and induces the change of the optical material reflective index, which will lead to optical axis deflection of the probe laser. We can extract the change of the weak signal using a position sensor and a lock-in amplifier, and then compute the absorption coefficient of the material on the pumping laser. A Schott fused silica coated metal thin film was used as the standard sample for absorption calibration. The system noise of the absorption test was about 0.4 ppm. The laser weak absorption test can also execute surface scanning to obtain the two-dimensional absorption distribution of a test region. In the test process, we executed surface scanning of a 3 mm × 3 mm test area with a 50 μm step size, for 3721 test points. In order to obtain statistically results, five regions with 3 mm × 3 mm were chosen randomly from each sample surface, the values were averaged to obtain the average absorption, and maximum of all tested regions is as peak absorption of the sample. The schematic of the photo-thermal deflection is shown in Fig. 13.

### Laser Damage Testing

**Figure 14:**
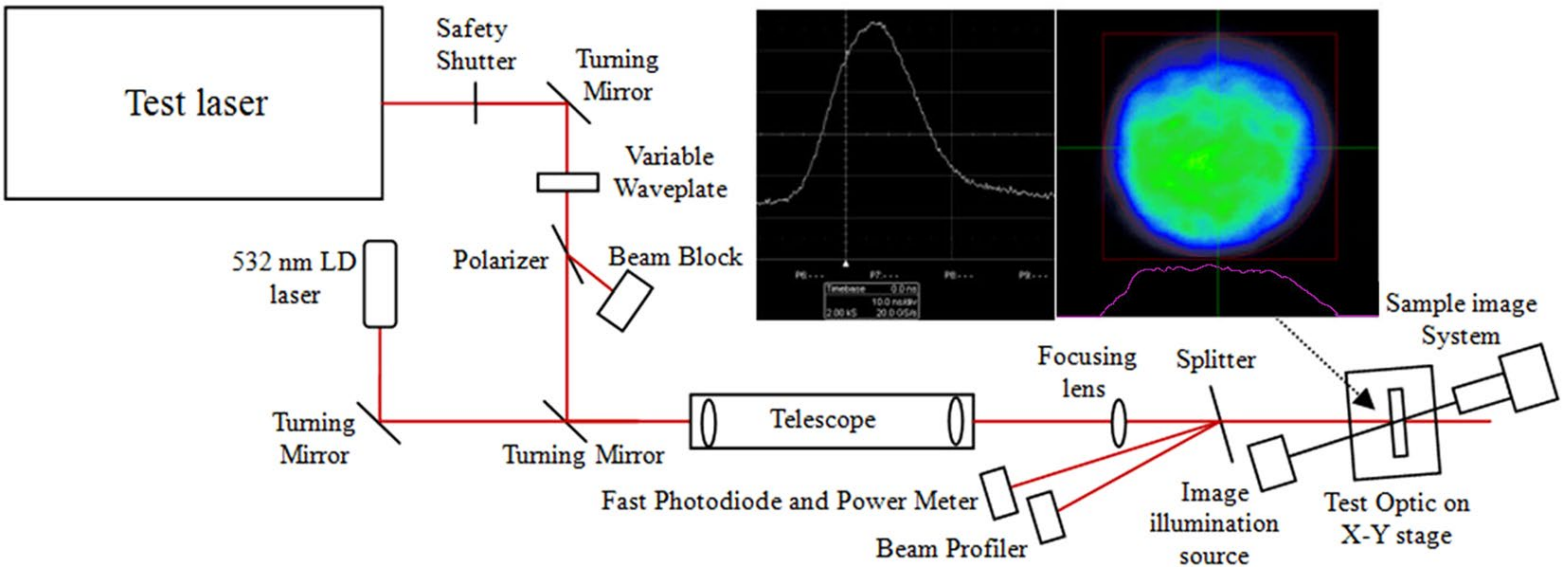
Layout of laser-induced damage test system and temporal and spatial profile of target plane.

**Figure 15:**
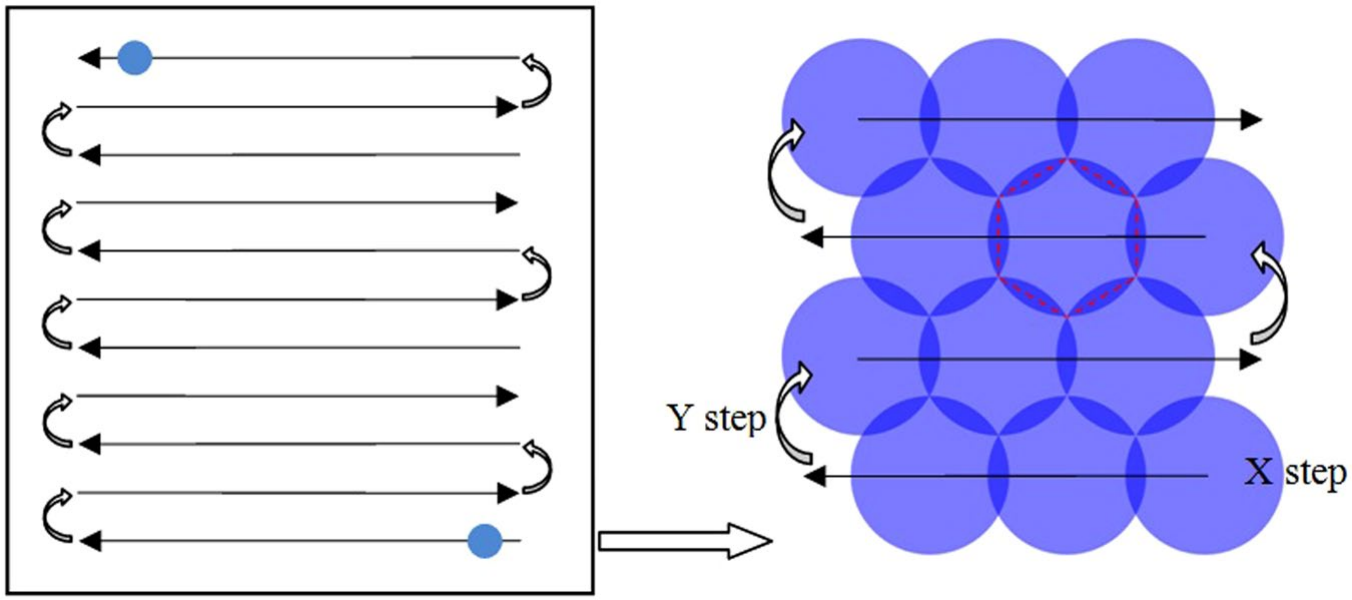
Schematic of Raster scan and regular hexagon beam overlap model.

The test laser is a tripled Nd:YAG system providing polarized 355 nm and a 9.3 ns pulse duration(FWHM) at 10 Hz pulse shoot repetition frequency. The pulse energy density (fluence) is set by a computer-controlled waveplate/polarizer assembly after the safety shutter. Turning mirrors direct the beam into a telescope and a focusing lens which produces a near flat-top beam with a 2.5 mm (1/e^2^) beam diameter. The sample imaging system is a digital optical microscopic with a 3.1 Megapixel CMOS USB 2.0 Camera, 90 mm of working distance and 50 X~400 X magnification. In the case of 100 X magnification, it can provide 2 μm resolution images of the sample surface immediately before and after each laser pulse. The layout of the laser-induced damage test system and the beam temporal and spatial profiler of the target plane is shown in Fig. 14. The measurement procedure of the laser-induced damage threshold followed the R on 1 method, which is implemented by ramping the fluence at every test point in a continuous ramp, with a starting fluence of 9 J/cm^2^ and a fluence increasement step of 1 J/cm^2^. By this method, zero probability damage thresholds were obtained by linear fitting of the relationship of test fluence on damage probability. We then used Raster-scan to test the damage densities with a 9 cm × 9 cm sampling area from all the samples(Fig. 15). A regular hexagon beam overlap model was used to evenly fill the whole sample surface, and a step size per shot of X and Y direction was computed according to the nominal configuration with a 2.5 mm 1/e^2^ beam diameter. The typical laser operation parameters for a large high-power laser facility is 3ω, 5 ns, and 8 J/cm^2^. Therefore, an 8 J/cm^2^ fluence of equivalent 5 ns was as energy density of damage density test, which is obtained by specifying the fluence important for initiation of damage on fused silica exit surfaces in terms of 5 ns Gaussian pulse equivalent^37^:

2 $${F}_{5nsEquiv}=F\times {(\frac{5.0}{\tau })}^{0.5}$$

Here $\tau$ is the Gaussian pulse width of the damage test laser, 9.3 ns. For every raster scanning pulse, the sample imaging system identifies all new damage points by comparing an image of the test area prior to the laser pulse with an image of the same area taken after the laser pulse. The damage density can be calculated when all new damage points in the whole sampling area have been counted.
